# Methylphenidate dose–response in children with ADHD: evidence from a double-blind, randomized placebo-controlled titration trial

**DOI:** 10.1007/s00787-023-02176-x

**Published:** 2023-03-02

**Authors:** Karen Vertessen, Marjolein Luman, James M. Swanson, Marco Bottelier, Reino Stoffelsen, Pierre Bet, Annemiek Wisse, Jos W. R. Twisk, Jaap Oosterlaan

**Affiliations:** 1 Clinical Neuropsychology Section, Amsterdam Public Health Research Institute, Vrije Universiteit Amsterdam, Amsterdam, The Netherlands; 2 Department of Child and Adolescent Psychiatry, University Psychiatric Centre KU Leuven, Leuven, Belgium; 3 Levvel Specialists in Youth and Family Care, Amsterdam, The Netherlands; 4 Department of Pediatrics, University of California, Irvine, USA; 5 Child Study Center Accare, UMC Groningen, Groningen, The Netherlands; 6 Department of Clinical Pharmacology and Pharmacy, Amsterdam UMC, VU Medical Center, Amsterdam, The Netherlands; 7 Youz, Center for Youth Menthal Healthcare, Velsen-Noord, The Netherlands; 8 Department of Epidemiology and Data Science, Amsterdam UMC, Amsterdam, The Netherlands; 9 Emma Neuroscience Group, Department of Pediatrics, Amsterdam Reproduction and Development, Emma Children’s Hospital, Amsterdam UMC, University of Amsterdam, Amsterdam, The Netherlands; 10 Child Study Group, Clinical Neuropsychology Section, Vrije Universiteit Amsterdam, Van der Boechorststraat, 7-9, 1081 BT Amsterdam, The Netherlands

**Keywords:** Attention-deficit, Hyperactivity disorder, Methylphenidate, Dose–response, Placebo-response

## Abstract

Methylphenidate (MPH) is highly efficacious in reducing symptoms of attention-deficit/hyperactivity disorder (ADHD) in children. Generally increased doses are found to result in better symptom control; however, it remains unclear whether this pattern can be observed at the individual level, given the large heterogeneity in individual dose–response relationships and observed placebo responses. A double-blind, randomized, placebo-controlled cross-over trial was used to compare weekly treatment with placebo and 5, 10, 15 and 20 mg of MPH twice daily on parent and teacher ratings of child ADHD symptoms and side effects. Participants were 5–13-year-old children with a DSM-5 diagnosis of ADHD (*N* = 45). MPH response was assessed at group and individual levels and predictors of individual dose–response curves were examined. Mixed model analysis showed positive linear dose–response curves at group level for parent and teacher rated ADHD symptoms and parent rated side effects, but not for teacher rated side effects. Teachers reported all dosages to improve ADHD symptoms compared to placebo, while parents only reported > 5 mg/dose as effective. At the individual level, most (73–88%) children, but not all, showed positive linear dose–response curves. Higher severity of hyperactive-impulsive symptoms and lower internalizing problems, lower weight, younger age and more positive opinions towards diagnosis and medication partly predicted steeper linear individual dose–response curves. Our study confirms that increased doses of MPH yield greater symptom control at a group level. However, large interindividual variation in the dose–response relationship was found and increased doses did not lead to greater symptom improvement for all children. This trial was registered in the Netherlands trial register (# NL8121).

**Supplementary Information:**

The online version contains supplementary material available at 10.1007/s00787-023-02176-x.

## Introduction

Attention-deficit hyperactivity disorder (ADHD) is the most diagnosed childhood-onset psychiatric disorder [1]. Methylphenidate (MPH) is highly efficacious in reducing ADHD symptoms, with effect sizes close to 1.0 [2]. International guidelines [3, 4] recommend ‘stepwise titration’ to determine the therapeutic dose in which treatment starts with a low dose and is gradually increased until the most effective dose with acceptable side effects [3–6]. Absolute rather than weight-based dosing is recommended, because body mass fails to predict optimal dose [7].

Currently, the efficacy of stepwise MPH titration is challenged by two issues. First, stepwise titration assumes that higher individual doses lead to greater ADHD symptom reduction in a linear manner. Several studies [6, 8, 9] have validated this assumption, showing that children who do not respond to lower doses have a higher probability of symptomatic improvement with increasing dose. However, studies focusing on individual outcomes [10–13] found that optimal doses varied between 10 and 50 mg/day and between 5 and 20 mg per administration, translating into a fourfold difference between the lowest and highest optimal dose. These findings cannot be explained by the weight of the child, given that small children (low in weight) may require relatively high doses, while more heavier children may require relatively small doses [13, 14]. Also differences in absorption and metabolism do not provide an explanation for the heterogeneity in optimal doses, since children who respond to low doses (5 mg per administration) may show low serum concentrations of MPH (4–5 ng/ml at *T*_max_) and those who respond to high doses (20 mg per administration) may show high serum concentrations (12–15 ng/ml) [13, 14]. Moreover, studies [15, 16] investigating individual dose–response relations reported nonlinear dose–response curves for the effects of MPH, implying that increased doses may not always lead to better symptom control [15, 16]. Taken together at a group level, increased doses of stimulants are associated with improved efficacy however we may question, that high doses always lead to the greatest ADHD symptom reduction at an individual level.

Second, stepwise titration does not offer a comparison with placebo. A recent meta-analysis by Faraone and colleagues [17] showed a significant improvement of ADHD symptoms under placebo treatment in controlled pharmacological trials with effect sizes ranging between 0.36 and 0.75 SMD, depending on the rater. In the Multimodal Treatment of Attention Deficit Hyperactivity Disorder Study (MTA study) 10% of children were classified as placebo responders, with these children showing no additional beneficial effects of MPH to placebo [18]. If no comparison with placebo is made, placebo responders are less likely to be identified and are exposed to MPH side effects without profiting from MPH specific effects.

An alternative method to investigate dose–response is double-blind, placebo-controlled titration (PCT). Different doses of MPH and placebo are prescribed double-blind and in random order. The effects of each dose are assessed in terms of symptom reduction and side effects [19]. Subsequently, it can be determined whether the child is a non-responder, placebo-responder or responder. For responders, the optimal dose provides most optimal symptom control with minimal side effects.

Another point that complicates MPH titration is the lack of clinically useful predictors for individual response. Studies aiming to predict children’s response to MPH, showed weaker responses to MPH in older children, children with more internalizing symptoms and in those with less severe symptoms of ADHD [20, 21]. However, most of these studies did not include the impact of dose on efficaciousness. One exception is the MTA study [12] that showed steeper dose–response curves for younger and lighter children, but did not confirm the effect of internalizing symptoms. Thus far little attention has been given to placebo-related effects. However, variables associated with placebo effects are important when investigating predictors for the dose–response relationship for MPH. Placebo effects have a prominent role in the overall effect of MPH [22, 23] and are highly related to patients expectations [24]. These expectations even lead to increases of synaptic dopamine, without MPH administration, which has been shown in a PET-study by Volkow et al. [23] Thus, variables measuring expectations and attitudes towards treatment could be interesting in predicting dose–response relations of MPH. Taken together, little is known about possible predictors of the dose–response relation of MPH that may facilitate more personalized MPH titration.

The aim of the current study was to gain more insight into the dose–response curves of MPH in terms of ADHD symptom reduction and side effects using PCT. The blinded, randomized and placebo-controlled titration trials offered the unique opportunity to compare dose–response curves at group and individual level. First, dose–response curves were studied at group level by determining the function best describing the dose–response relationship and comparing the effects of doses of 5, 10, 15 and 20 mg and placebo. Likewise, individual dose–response curves were determined. Finally, variables that showed promise in clinically predicting response to MPH, were explored as possible predictors of the individual dose–response curves.

## Methods

### Study design

Children were recruited from mental health clinics in The Netherlands between May 2017 and December 2019. Inclusion criteria were as follows: (a) a clinical diagnosis of ADHD according to DSM-5, (b) 5–13 years of age, (c) IQ > 70, (d) indication for MPH treatment, as determined by the treating physician, and (e) no pharmacological treatment for ADHD 4 weeks prior to study entry. Comorbid diagnoses were not an exclusion criterion. Diagnostic status was confirmed by the first author (K.V.) using the (1) Kiddie–Schedule for Affective Disorders and Schizophrenia for School-Age Children–Present and Lifetime Version (K-SADS), a semi-structured standardized, investigator-based parent interview [25] and (2) teacher rated Disruptive Behavior Disorder rating scale (DBDRS) assessing the presence and severity of symptoms of ADHD [26].

The PCT protocol was based on the titration protocol used in the MTA study [18], modified to improve clinical usability by weekly instead of daily dose changes [27]. All participants received the following treatment conditions: placebo and 5, 10, 15 and 20 mg of MPH twice daily (20 mg only for children > 25 kg [28] in a semi-randomized order. The randomization and blinding procedure is described in more detail in Supplement S1. We used dosing twice daily versus three times daily as this is the dominant Dutch clinical practice. The titration procedure started with a lead-in phase [29], consisting of 4 days in which all doses were administered in ascending order. If a dose was not tolerated it was excluded from the PCT. Duration of the PCT was 3 to 5 weeks, depending on the child’s weight and MPH doses tolerated. During PCT, treatment with a particular dose started on a Saturday and was administered for seven consecutive days, twice daily, at breakfast (around 8 a.m.) and at lunch time (around 12 a.m.).

At baseline and at the end of each week, parents and teachers completed the Strength and Weakness of ADHD symptoms and Normal Behavior Rating Scale (SWAN) [30] rating scale and an adapted version of the MTA Side Effect Rating Scale (see description below) [19]. The local ethics committee approved the study (METC VUMC, # 2016.594 & Netherlands trial register # NL8121).

### Outcome variables

#### ADHD symptoms

ADHD symptom severity was measured with the SWAN [30], adapted to measures symptom severity in the past week. This questionnaire contains the following two scales: the Inattention scale and the Hyperactivity/Impulsivity scale, each comprising nine items based on the DSM-IV symptoms of ADHD. Items are scored on a 7-point Likert scale ranging from − 3 to 3, with lower ratings reflecting worse symptoms. Items reflect both ends (strong and weak) of the behavior captured in each symptom. Therefore, it has the potential to reveal clearer and additional dose–response effects due to assessment across the full range of positive and negative manifestations of the behavior underling the symptoms of ADHD [31].

#### Side effects

Side effects were reported using an adapted version of the MTA Side Effect Rating Scale [19], using a scoring system according to Wigal [32]. Commonly reported side effects were rated on a 4-point scale (0 = *not at* all, 1 = *just a little*, 2 = *pretty much* and 3 = *very much*) measuring side effect severity in the past week. The total score was used in the analyses.

#### Predictors of individual MPH dose–response curves

To explore clinically useful predictors, a range of variables that have been associated with dose–response effects [12, 20, 21, 24] and can be routinely identified in standard clinical practice, were assessed at baseline. Candidate predictors included clinical characteristics, demographic variables and attitudes towards ADHD diagnoses and treatment and are described in the Supplement S2 and S3.

### Statistical analyses

Analyses were performed using STATA (version 16.0). To examine the effects of MPH dose on ADHD symptoms and side effects, mixed model analyses were conducted given the hierarchical data-structure. To investigate the dose–response curves at group level, two analyses were performed. First, to determine the function best describing the dose–response relationship linear dose–response curves were fitted as function of absolute dose (mg) for each of the following six outcome measures: parent- and teacher-rated inattention symptoms, hyperactivity-impulsivity symptoms and side effects. Next, it was evaluated if a second-, or third order polynomial better described the relationship by adding a quadric and S-shaped component, respectively. Additionally, the analysis were repeated with relative dose (mg/kg). Second, we treated dose as a categorical variable, comparing the effects of 5, 10, 15, and 20 mg (children > 25 kg only) on each of the six outcomes with a mixed model analysis.

As linear dose–response curves provided the best description of the dose–response curves at the group level, linear dose–response curves were also fitted to the individual dose–response curves. The regression coefficient of these individual dose–response curves was used to quantify the dose–response relationship for each outcome measure. Positive β-values indicate that increased doses of MPH were related to steeper linear dose–response curves, with increased doses resulting in larger reductions in ADHD symptoms or a smaller increase in side effects. Non-positive β-values indicate that higher MPH doses were related to more shallow linear dose–response curves, with increased doses resulting in smaller reductions in ADHD symptoms or a smaller increase in side effects.

Finally, the possibility to predict the variation in the individual linear dose–response curves, using variables assessed at study entry was explored. Mixed model analyses, with univariate preselection of significant predictors and backward selection procedure, were used to construct prediction models. Complying with the convention in this type of analysis, the threshold for significance was set at *p*-values < 0.10 [33]. The amount of variance explained (R.^2^) was calculated, with 1%, 9% and 25% used as the thresholds for small, medium and large effects, respectively [34].

## Results

### Sample

Forty-one clinicians from 13 youth mental health clinics across the Netherlands participated. Forty-five children were included in the analyses. Table 1 displays the participants’ demographic and clinical characteristics. One child did not receive the 15 and 20 mg due to a combination of severe side effects and dosing restriction (< 25 kg), eight children did not receive the 20 mg dose, six because of the dosing restrictions (< 25 kg) and two due to severe side effects. All clinicians fully adhered to the protocol with each tested MPH dose being prescribed for seven consecutive days, twice daily.

**Table 1:** Demographic and clinical characteristics of participating children (*n* = 45)

	M	SD	Min	Max
Age (years)	9.53	1.70	5.91	12.77
Sex (% male)	67%			
Weight (kg)	32.7	8.41	18	64
KSADS				
Inattention symptoms	7.07	1.62	0	8
Hyperactivity-impulsivity symptoms	5.84	2.29	2	9
ODD symptoms	2.11	2.24	0	8
SWAN inattention				
Parent	− 14.30	6.59	− 23	4
Teacher	− 13	6.02	− 26	10
SWAN hyperactivity-impulsivity				
Parent	− 13.42	7.58	− 27	17
Teacher	− 9.49	10.62	− 26	13
CBCL internalizing problems	57.49	9.98	33	74
Opinion on treatment (child)	4.35	1.67	1	9
Aversion towards medication (child)	1.60	0.79	1	3
Agreement with therapy				
Parent	17.91	2.73	11	20
Teacher	18.14	2.46	11	20
Treatment expectations				
Parent	4.25	0.81	2	5
Teacher	4.08	0.73	3	5

### Dose–response curves at group level

Results of the mixed model analyses on the dose–response curves (with absolute dose) at group level are depicted in Tables 2, 3 and 4. Regression coefficients of the second- and third order polynomial were non-significant (data available from the author). The relationship best describing the dose–response was a positive linear relationship for MPH dose and both parent and teacher ratings on the SWAN scales Inattention and Hyperactivity/Impulsivity, indicating that higher MPH doses resulted in a larger improvements in ADHD symptoms. For side effects measured with the parent-reported Side Effect Rating Scale, the relationship was best described by a positive linear effect, indicating that increased doses resulted in more side effects as observed by parents. No significant relationship was found between MPH dose and teacher-reported side effects. Additional analysis with relative dose (mg/kg) resulted in similar results (data available from the author).

**Table 2:** Effects of MPH on inattention, hyperactivity/impulsivity and side effects

	Placebo	5 mg	10 mg	15 mg	20 mg
	M (SD)	M (SD)	M (SD)	M (SD)	M (SD)
SWAN inattention					
Parent	− 8.93 (8.89)	− 9.53 (7.83)	− 6.16 (7.78)	− 4.86 (9.40)	− 4.54 (8.04)
Teacher	− 12.27 (7.17)	− 9 (7.18)	− 6.88 (9.08)	− 4.3 (9.41)	− 4.41 (7.25)
SWAN hyperactivity/impulsivity					
Parent	− 8.48 (8.58)	− 8.84 (8.46)	− 5.2 (6.70)	− 4.34 (8.66)	− 2.97 (7.86)
Teacher	− 11.46 (8.02)	− 7.19 (9.53)	− 4.90 (10.89)	− 2.88 (9.13)	− 3.34 (8.18)
Side effects					
Parent	4.98 (4.03)	6.07 (4.70)	5.64 (3.95)	6.86 (4.81)	5.79 (4.06)
Teacher	2.71 (3.03)	3.35 (2.85)	2.75 (3.16)	3.35 (3.54)	2.63 (2.63)

SWAN scores range from − 27 to 27, Side effects scores range from 0 to 39

**Table 3:** Dose–response curves fitted as function of dose at group level

Outcome	Linear trend
	Coef. (95% CI)
SWAN inattention	
Parent	0.29*** (0.15 to 0.42)
Teacher	0.42*** (0.28 to 0.55)
SWAN hyperactivity/impulsivity	
Parent	0.33*** (0.19 to 0.46)
Teacher	0.43*** (0.29 to 0.56)
Side effects	
Parent	0.08* (0.01 to 0.15)
Teacher	0.01 (− 0.04 to 0.05)

*p < 0.05; **p < 0.01; ***p < 0.001

Next, we treated dose as a categorical variable comparing the effects of each of the MPH doses and placebo on the six outcomes. Parents reported significant improvement with MPH doses of 10, 15 and 20 mg compared to placebo on the SWAN hyperactivity/impulsivity scale and for 15 and 20 mg doses on the SWAN inattention scale. Additionally parents reported significantly higher scores on the Side Effect Rating Scale for 15 mg compared to placebo. Teachers reported significant improvement on both SWAN scales for all MPH doses compared to placebo. For side effects measured with the teacher-reported Side Effect Rating Scale, none of the MPH doses differed significantly from placebo. None of the other comparisons between the tested MPH doses was significant.

### Dose–response curves at individual level

**Fig. 1:**
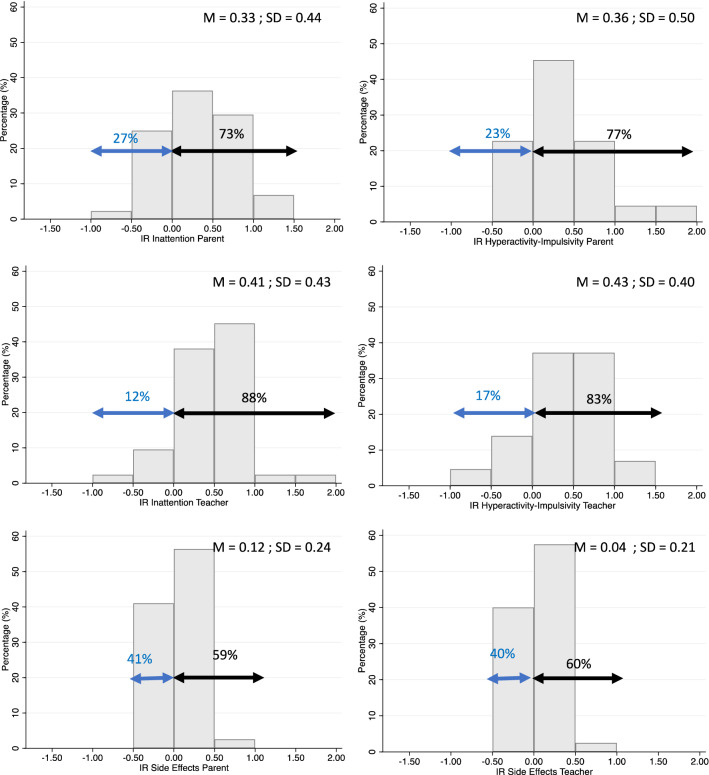
Histogram of regression coefficients obtained from the individual linear dose–response curves. The left arrows represent the percentage of children with negative dose–response curves, while the right arrows represent the percentage of children with positive dose–response curves

To investigate individual response to MPH, a linear trend was fitted to the individual dose–response curves. The regression coefficients of these linear curves were used to quantify the individual dose–response relationships. Figure 1 shows the distribution of the regression coefficients for each of the six outcome measures. In line with the findings at group level, for parent- and teacher-reported inattention and hyperactivity/impulsivity on the SWAN, the majority of subjects (73–88%), showed positive regression coefficients. However, 12 to 27% of the dose–response curves yielded non-positive regression coefficients, indicating that in these children, higher MPH doses did not result in lower levels of ADHD symptoms.

**Table 4:** Effects of different MPH doses versus placebo

Outcome	5 mg	10 mg	15 mg	20 mg
	Coef. (95% CI)	Coef. (95% CI)	Coef. (95% CI)	Coef. (95% CI)
SWAN inattention				
Parent	− 0.62 (− 3.46 to 2.23)	2.76 (− 0.08 to 5.61)	3.93** (1.07 to 6.80)	4.82** (1.75 to 7.88)
Teacher	3.22* (0.39 to 6.05)	5.06** (2.20 to 7.93)	7.36*** (4.45 to 10.27)	8.20*** (5.05 to 11.36)
SWAN hyperactivity/impulsivity				
Parent	− 0.31 (− 3.08 to 2.47)	3.34* (0.56 to 6.11)	4.18** (1.38 to 6.97)	5.91*** (2.92 to 8.90)
Teacher	3.91** (1.15 to 6.68)	6.10*** (3.32 to 8.90)	7.76*** (4.95 to 10.57)	8.52*** (5.52 to 11.53)
Side effects				
Parent	1.16 (− 0.27 to 2.61)	1.03 (− 0.48 to 2.53)	2.01** (0.52 to 3.55)	1.40 (− 0.24 to 3.04)
Teacher	0.48 (− 0.49 to 1.45)	0.02 (− 0.93 to 0.98)	0.87 (− 0.10 to 1.84)	− 0.08 (− 1.09 to 0.94)

The regression coefficient represents the difference in the outcome between the MPH dose and placebo

*p < .0.05; **p < 0.01; ***p < 0.001

For both parent- and teacher-reported side effects, the majority of subjects (59 and 60%, respectively) showed positive regression coefficients, indicating greater severity of side effects with increased doses. This indicates that 40–41% of the children did not show an increase in side effects when increasing the dose. Note that for both parent- and teacher-reported side effects less than 10% of the children show beta coefficients > 0.5, suggesting that most children showed shallow dose-side effect curves.

### Prediction models for regression coefficients of linear dose–response

Table 5 shows the prediction models. Higher child-reported aversion towards medication was significantly related, with a medium effect (*R*^2^ = 17%), to a shallower linear dose–response curves for parent-reported inattention on the SWAN. Higher parent-reported KSADS hyperactivity-impulsivity was significantly related, with a medium effect (*R*^2^ = 14%), to steeper linear dose–response curves for parent-reported hyperactivity-impulsivity on the SWAN. Higher Child Behavior Checklist (CBCL) Internalizing symptoms and lower parent- and teacher*-*reported agreement with diagnosis and therapy were significantly related to shallower linear dose–response curves for teacher-reported inattention and hyperactivity-impulsivity on the SWAN, with large effects (respectively *R*^2^ = 27% and 48%). Regarding side effects, older age was significantly related, with a medium effect (*R*^2^ = 9%), to shallower linear dose–response curves for parent-reported side-effects. Lower weight and lower CBCL internalizing symptoms were significantly related, with medium effects (*R*^2^ = 28%), to shallower linear dose–response curves for teacher-reported side-effects.

**Table 5:** Results of the regression analysis testing the relationship between demographic and clinical characteristics and coefficients of the linear individual dose–response curves

Outcome	Predictors	Multivariable results	Variance explained
SWAN inattention			
Parent	Aversion towards taking medication (child)	β(SE) = − 0.22 (0.08), *p* = 0.004	*R*^2^ = 17%
Teacher	Teacher-reported agreement with diagnosis and therapy	β(SE) = 0.05 (0.02), *p* = 0.03	*R*^2^ = 27%
	CBCL internalizing problems	β(SE) = − 0.01 (0.01), *p* = 0.06	
SWAN hyperactivity/impulsivity			
Parent	KSADS hyperactivity-impulsivity	β(SE) = 0.08 (0.03), *p* = 0.009	*R*^2^ = 14%
Teacher	CBCL internalizing symptoms	β(SE) = − 0.01 (0.01), *p* = 0.01	*R*^2^ = 48%
	Parent-reported agreement with diagnosis and therapy	β(SE) = 0.05 (0.02), *p* = 0.02	
	Teacher-reported agreement with diagnosis and therapy	β(SE) = 0.05 (0.02), *p* = 0.02	
Side effects			
Parent	Age	β(SE) = − 0.04 (0.02), *p* = 0.08	R^2^ = 9%
Teacher	Weight	β(SE) = − 0.01 (0.00), *p* = 0.06	R^2^ = 28%
	CBCL internalizing symptoms	β(SE) = − 0.01 (0.00), *p* < 0.001	

Negative β-values indicate that a higher score on the predictor was related to a more shallow linear dose–response curve, whereas positive β-values indicate that a higher score on the predictor was related to a steeper linear dose–response curve. *R*^2^ = amount of variance explained

For all analyses, sensitivity analyses were conducted, excluding children with clinical scores on the internalizing scale of the parent reported Child Behavior Checklist (CBCL) because internalizing symptoms might be perceived as ADHD-symptoms and therefore might have biased our results. This did not change our main findings (data available from the author).

## Discussion

This study aimed to gain more insight into dose effects of MPH on ADHD symptoms and side effects using a double-blinded, randomized, placebo-controlled crossover design. Our results demonstrated that, at group level, the dose–response relationship for parent- and teacher-rated ADHD symptoms can best be described as a positive linear relationship, indicating that higher MPH doses resulted in a larger improvements in ADHD symptoms. Regarding side effects, parents reported increased doses to result in more side effects, although this trend was not observed by teachers. Comparisons of the effects of placebo and the tested MPH doses showed that doses of 10 mg and higher yielded significant reductions in parent-reported ADHD symptoms, whereas all tested doses yielded significant reductions in teacher-reported ADHD symptoms. Regarding side effects, parents reported more side effects with doses 15 mg compared to placebo, whereas teachers observed no differences between any of the MPH doses and placebo. Examining individual dose–response curves showed that a significant part (12–27%) of children did not show a positive linear dose–response curve. For side effects, only 59–60% of children showed an increase of side effects with increasing doses. Explorative analyses showed that higher severity of hyperactive-impulsive symptoms and lower internalizing problems, lower weight, younger age and more positive opinions towards diagnosis and medication contributed to predicting steeper linear individual dose–response curves.

The positive linear dose–response curves observed at group level and the finding that only high doses show parent-reported symptom reduction are consistent with the idea that increased doses of MPH lead to a larger reductions in ADHD symptoms. However, our results clearly demonstrate that a significant subgroup does not show larger symptom reduction with increased doses. Thus, the current study replicates that the effects of MPH on ADHD symptoms can be best described as a positive linear dose–response relationship, but also shows that divergent responses to dose increase are not incidental findings.

At group level, higher MPH doses were related to more parent-reported, but not to higher teacher-reported side effects. This is in line with the findings from the MTA-study that showed parents to report more dose-related adverse effects, making them the best reporters of side effects [19]. For the majority of subjects, increased doses of MPH are related to greater severity in side effects, although this inverse relationship between dose and side effects was not observed in a substantial part of the children (39–40%). For most children our data support the advice to explore the full range of MPH doses [7], with a low risk of clinical important side effects.

This study explored predictors for the individual dose–response to MPH. To the best of our knowledge, the current study is the first to explore the role of attitudes towards diagnosis and medication in the response to MPH. Interestingly, we found that higher parent and teacher agreement with the diagnosis and therapy were related to larger improvements in teacher-reported ADHD symptoms with increasing dose. Furthermore, larger aversion of the child towards taking medication, was related to smaller improvements in ADHD symptoms with increasing dose. There are two possible explanations for these findings. On the one hand this suggests that negative attitudes towards the diagnosis and medication may result in smaller placebo effects, which in turn may diminish the beneficial effects. On the other hand it’s also possible that negative attitudes towards diagnosis and medication may result in more accurate ratings of ADHD symptoms following pharmacological intervention, because these ratings are less biased by the expectation of symptom improvement.

Additionally, more severe hyperactivity–impulsivity symptoms and lower internalizing problems were associated with a larger reduction of ADHD symptoms with increasing MPH doses. This is in line with previous research [20, 21, 35] showing these measures to be associated with a larger response to MPH regardless of dose. In contrast with the results from the MTA-study [12], age was not significantly related to the dose–response to MPH. Side effects, higher age, weight and internalizing symptoms, were related with more shallow dose response curves, and thus a smaller increase of side effects with increasing dose.

Our findings should be viewed with some limitations in mind. First, our results only pertain to the short-term effects of MPH dose. Follow-up from the MTA-study has shown that initial titration does not prevent the need for subsequent maintenance adjustments; however, end-of-titration optimal dose and maintenance dose were strongly related [10]. Second, no instruments were used to report treatment fidelity. Children who had greater aversion to taking medication may not have taken medication as prescribed which, in turn, could affect symptom changes. Third, MPH was administered twice daily, in accordance with dominant Dutch clinical practice. The observed differences between dose–response effects for teacher and parent ratings might be affected by the time–response effects and the time of assessment by observations during the day (by teachers) and after school (by parents). Fourth, only the clinical diagnosis of ADHD and symptoms of ODD were assessed using standardized assessments conducted by the researchers. No standardized assessment of other comorbid disorders was conducted. Consequently, we were not able to test the possible predictive power of comorbidity for dose–response effects

Despite these limitations our study is one of few studies that attempted to analyze dose–response curves of MPH in ADHD. Last, we explored predictors for individual dose response as a first attempt to study what dosage works for whom. We explored multiple potential predictors in a relatively small sample, which generated important hypotheses regarding the response to MPH. However, due to limited power, weaker predictors may have remained undetected. Further research is needed to replicate these findings and draw stronger conclusions regarding subgroups that respond differently to MPH dosages, such as subgroups that differ in attitudes towards diagnoses and treatment for ADHD.

## Conclusion

Although increased doses of MPH yield greater symptom control at a group level, there is large inter-individual variation in the dose–response relationship. As dose–response curves can only be partly explained based on patient characteristics, our findings emphasize the need to use an objective evaluation of the full range of the possible therapeutic doses for all individual patients as substantiated in the MTA-study [18] and in the recent meta-analyses by Farhat and colleagues [6]. Side effects should be monitored when increasing the dose [6], although exploring the clinical effect of the full dose range is often possible without increasing side effects. Physicians should be mindful about attitudes towards the diagnosis and medication as they might contribute to MPH treatment effects.

### Supplementary Information

Below is the link to the electronic supplementary material.

Supplementary file1 (DOCX 46 KB)

## Data Availability

Deidentified individual participant data (including data dictionaries) will be made available, upon publication to researchers who provide a methodologically sound proposal for use in achieving the goals of the approved proposal. Proposals should be submitted to k.vertessen@vu.nl.

## Abbreviations

ADHD: Attention-deficit/hyperactivity disorder
MPH: Methylphenidate
K-SADS: Kiddie–schedule for affective disorders and schizophrenia for school-age children present and lifetime version
DBDRS: Disruptive behavior disorder rating scale
PCT: Placebo controlled titration

## Code availability

The statistical analysis plan and codes will be made available upon request. Proposals should be submitted to k.vertessen@vu.nl.
