# Supplementary material for: Methylphenidate dose–response in children with ADHD: evidence from a double-blind, randomized placebo-controlled titration trial
Source: Eur Child Adolesc Psychiatry. 2023 Mar 2;33(2):495–504. doi: 10.1007/s00787-023-02176-x (PMC10869379; doi:10.1007/s00787-023-02176-x)
Supplement: Supplementary file 1 — Supplementary file1 (DOCX 46 KB) [file 787_2023_2176_MOESM1_ESM.docx]

**Supplement: Methylphenidate dose-response in children with ADHD:**

**Evidence from a double-blind, randomized placebo-controlled titration trial**

### **Blinding and Randomization………………………………………………………..…p2**

### **Predictors of Individual MPH Dose-Response Curves ………………………………p2**

1. **Custom-made questionnaires**

3.1 Agreement with the therapy……………………………………………………...…p3

3.2 Treatment expectations………………………………………………………..……p3

3.3 Opinion on diagnosis and treatment………………………………………….….…p3

3.4 Aversion towards medication…………………………...……………………….…p4

1. **Blinding and Randomization**

Prior to study initiation randomization spreadsheets for medication order were generated using a computerized random number generator [1] The order of the doses condition differed between children, and was implemented randomly with restrictions that the highest dose was never the starting dose or the dose after the placebo week.[2] Randomization occurred by collaborators from the academic pharmacy who had no contact with participants. The dose order was blinded for participants and treating physician until the end of the titration period. All placebo and MPH tablets, appeared identical and were specifically produced by Tiofarma (Oud-Beijerland, The Netherlands, license 2165-F) for this study under European Union Good Manufacturing Practice annex 13 guidelines and appeared identical in color and shape.

1. **Predictors of Individual MPH Dose-Response Curves**

In order to determine clinically useful predictors of individual dose-response functions a range of demographic and clinical variables, that would be available in standard clinical practice, were assessed at baseline. Sex, IQ, age (years) and weight (kg) were reported by the physician. ADHD symptom severity was assessed with the ADHD scales of the K-SADS[3] and the parent and teacher ratings on the SWAN[4] scales Inattention and Hyperactivity/Impulsivity. Oppositional defiant disorder (ODD) severity was assessed with the ODD scale of the K-SADS[3]. Internalizing behavior was measured with the Internalizing Problems scale of the parent reported Child Behavior Checklist (CBCL). Custom-made parent and teacher questionnaires were used to assess ‘Agreement with the diagnosis and therapy’ and ‘Treatment expectations’ with higher scores indicating higher agreement and higher expectations. Finally, a custom-made child questionnaire was used to assess the child’s ‘Opinion on diagnosis and treatment’ and ‘Aversion towards medication’, higher scores indicating a more negative opinion towards diagnosis and treatment and a higher Aversion towards medication.

1. **Custom-made questionnaires**

3.1 Agreement with the diagnosis and therapy

Items were scores on a 5-point scale: 1=not at all, 2=not entirely, 3=neutral, 4=a little, 5= completely. The total score of all questions combined was used, Cronbach’s Alpha for parent = .74 and teacher = .82.

Questions used were:

*Do you support the ADHD diagnosis made?*

*Do you support the general approach to treatment?*

*Do you support the treatment with medication?*

*Do you have confidence in the method by which the dose of the drugs will be determined?*

3.2 Treatment expectations

Items were scores on a 5-point scale: 1=not at all, 2=not entirely, 3=neutral, 4=a little, 5= completely. The total score was used.

Question used was:

*How much do you expect ADHD symptoms to improve?*

3.3 Opinion on diagnosis and treatment

Items were scores on a 3 point scale: 1=no , 2=a little , 3=yes. The total score of all questions combined was used, Cronbach’s Alpha = .70

Questions used were:

*- Do you dislike having ADHD?*

*- Are you ashamed of your ADHD diagnosis?*

*- Are you ashamed of your medications?*

3.4 Aversion towards medication

Items were scores on a 3 point scale: 1=no , 2=a little , 3=yes. The total score was used.

Questions used were:

*- Do you dislike taking medication?*

**References**

[1] G. Dallal, “Randomization plan generator; first generator.” 2007.

[2] T. W. P. Janssen, M. Bink, K. Geladé, R. van Mourik, A. Maras, and J. Oosterlaan, “A Randomized Controlled Trial Investigating the Effects of Neurofeedback, Methylphenidate, and Physical Activity on Event-Related Potentials in Children with Attention-Deficit/Hyperactivity Disorder,” *Journal of Child and Adolescent Psychopharmacology*, vol. 26, no. 4, pp. 344–353, May 2016, doi: 10.1089/cap.2015.0144.

[3] Reichart CG, Wals M, and Hillegers M, “Vertaling K-sads.” HC Rümke Groep, Utrecht, 2000.

[4] A. B. Arnett *et al.*, “The SWAN Captures Variance at the Negative and Positive Ends of the ADHD Symptom Dimension,” *Journal of Attention Disorders*, vol. 17, no. 2, pp. 152–162, Feb. 2013, doi: 10.1177/1087054711427399.
